# Remarkable Synergy When Combining EZH2 Inhibitors with YM155 Is H3K27me3-Independent

**DOI:** 10.3390/cancers15010208

**Published:** 2022-12-29

**Authors:** Jun Yang, Andrew M. Davidoff

**Affiliations:** 1 Department of Surgery, St. Jude Children’s Research Hospital, Memphis, TN 38105, USA; 2 St. Jude Graduate School of Biomedical Sciences, St. Jude Children’s Research Hospital, Memphis, TN 38105, USA; 3 Department of Pathology and Laboratory Medicine, College of Medicine, The University of Tennessee Health Science Center, Memphis, TN 38163, USA; 4 Division of Pediatric Surgery, Department of Surgery, University of Tennessee Health Science Center, Memphis, TN 38105, USA

**Keywords:** EZH2, BIRC5, YM155, EPZ5687, EPZ6438, GSK343, PRC2, H3K27me3, neuroblastoma

## Abstract

**Simple Summary:**

Systems biology analysis of gene expression across solid tumors identifies a 27 signature that centers on EZH2, the key component of polycomb repressive complex 2. Our study demonstrates that targeting the 27 gene network by the combination of BIRC5 and EZH2 inhibitors achieves remarkable anticancer synergy that is independent of the histone methyltransferase activity of EZH2.Globlal gene expression analysis in combination with pathway analysis shows that combination of YM155, the BIRC5 inhibitor, with EZH2 inhibitors induces unfolded protein response.

**Abstract:**

Targeting multiple molecules in the same biological network may maximize therapeutic efficacy. In this study, we identified a 27-gene module that is highly expressed in solid tumors, encoding actionable targets including EZH2 and BIRC5. The combination of EZH2 inhibitors and a BIRC5 inhibitor, YM155, results in a remarkable synergistic effect. The action of EZH2 inhibitors in this process is independent of the histone methyltransferase activity of polycomb repressive complex 2. Our study reveals a potential therapeutic approach for treating solid tumors by simultaneously targeting EZH2 and BIRC5.

## 1. Introduction

Neuroblastoma is a solid tumor arising from aberrant growth of the developing sympathoblast lineages of neural crest cells [1,2,3,4,5,6]. Neuroblastoma is the most common type of cancer in infants and accounts for 15% of deaths from childhood cancer [7]. Although outcomes for children with low- or intermediate-risk disease are excellent, those for children with high-risk disease remain less than 50% [8]. Hence, there is a pressing need to identify novel therapeutic targets for neuroblastoma. While neuroblastoma is heterogeneous in cellular and transcriptomic states [9,10,11], amplification of the *MYCN* oncogene is one of the most significant biological features of high-risk neuroblastoma [12]. Loss of heterozygosity at chromosome 1p36 or 11q23 also occurs frequently and is associated with poor survival [13]. Next-generation sequencing (NGS) studies have revealed few recurrent somatic mutations (e.g., *ALK*) at the time of diagnosis in neuroblastoma patients [14,15]. These findings suggest that targeted therapy based on molecular attributes that predict therapeutic outcome may benefit only some patients. Recent preclinical studies have revealed multiple promising therapeutic targets and therapies [16,17,18,19,20,21,22,23]. However, considering the clinical challenges of treating high-risk patients, it is important to understand the biological functions and pathways in neuroblastoma for the development of additional targeted approaches.

Polycomb group proteins (PcG) are transcriptional repressors that form the polycomb repressive complexes PRC1 and PRC2 to epigenetically silence gene expression [24]. PRC2 consists of 4 core proteins (EZH2, SUZ12, EED, and RbAp46/48) that are conserved from *Drosophila* to mammals [25]. Although each component of PRC2 is important for function, the histone methyltransferase EZH2 is crucial as it adds repressive histone methyl marks on lysine 27 of histone H3 (H3K27), as well as non-histone proteins such as GATA4 [26], JARID2 [27], RORa [28], STAT3 [29], and TALIN [30]. PRC2 is involved in various biological processes such as proliferation, differentiation, cell identity maintenance, stem cell plasticity, and cancer [25]. Recurrent somatic mutations of EZH2 have been identified in subtypes of lymphoma, and EZH2 gain-of-function mutations alter substrate specificity for promoting hypertrimethylation of H3K27 [31]. However, the identification of loss-of-function mutations in PRC2 components in leukemia and in malignant peripheral nerve sheath tumors indicates its tumor suppressive function [32,33,34]. Targeting PRC2 function (mainly by inhibiting EZH2 with small molecules) for cancer treatment has been extensively studied, and specific inhibitors have been developed for testing in clinical trials [35]. EZH2 is important for neuroblastoma cell survival by suppressing differentiation and tumor suppressors and for stabilizing the MYC oncoprotein [36,37,38,39,40], indicating that targeting EZH2 could be an alternative approach for neuroblastoma treatment. However, it remains unknown what combination therapies could maximally enhance the efficacy of EZH2 inhibitors,

PRC2 expression is regulated by the retinoblastoma protein pRB-E2F pathway [41]. Previous studies have shown that miRNAs are involved in PRC2 regulation. For example, EZH2 is targeted by miR-101 [42]. Despite extensive studies, the functions of PRC2, especially with regard to EZH2 in cancer, are not entirely clear. Comet et al. proposed that PRC2 functions to maintain cell identity in cancer [43]. However, the oncogenic function of EZH2 in cells of castration-resistant prostate cancer seems to be independent of PRC2-mediated transcriptional repression [44]. This functional switch is dependent on the phosphorylation of EZH2 and requires an intact methyltransferase domain [44]. Interestingly, studies show that EZH2 is phosphorylated during the G2/M phase of the cell cycle by CDK1 and CDK2 [45,46,47], suggesting its function in cell cycle regulation. EZH2 can also methylate STAT3 and increase its activity in glioblastoma stem cells [29,48]. Furthermore, EZH2 has a methyltransferase activity-independent function in stabilizing the spindle assembly checkpoint protein BubR1 during meiotic maturation of mouse oocytes [49]. In this study, we identified a 27-gene signature centered on EZH2 across many types of cancers, including neuroblastoma, which is targeted by miR-34. Most of the gene products in this signature (e.g., MELK, TTK, BIRC5, and AURKA) regulate mitosis. The combination of EZH2 inhibitors and YM155, a BIRC5 (also called survivin) inhibitor, led to remarkable synergistic activity, which is independent of PRC2-mediated H3K27 methylation. These findings suggest a novel mechanism of action for EZH2 inhibitors that can be exploited to develop new combination therapies. 

## 2. Results

### 2.1. Tumor Suppressor miR-34 Regulates the Expression of EZH2

**Figure 1:**
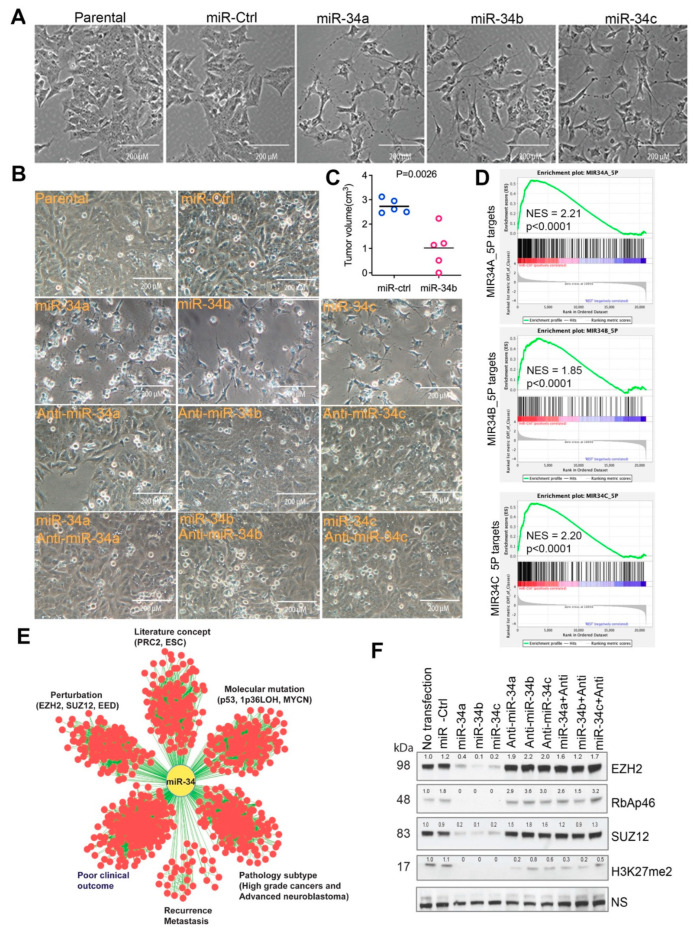
miR-34 is tumor suppressive and targets multiple gene signatures and PRC2 complex. (**A**) Neurite outgrowth after 72 h transfection of miR-34 paralogs into BE2C cells. Photos were taken under a microscope (magnification, 20×). (**B**) Cell morphology change after 72 h transfection of BE2C cells with miR-34 and anti-miRs. Photos were taken under microscope (20×). (**C**) Tumor volume in BE2C xenografts (n = 5) with lentiviral-mediated miR-34b expression. (**D**) Gene set-enrichment analysis results for the genes downregulated by miR-32 targets (miR-34A-5P normalized enrichment score (NES) = 2.21, *p* < 0.0001; mir-34B-5P NES = 1.85, *p* < 0.0001; miR-34C-5P NES = 2.20, *p* < 0.0001). (**E**) Concept analysis of miR-34 signature derived from BE2C cells. The association of the signatures with *p* < 0.000001 and odds ratio >2 was presented by Cytoscape program. (**F**) Western blotting for PRC2 components with indicated antibodies after 72 h transfection of miR-34 and anti-miRs into BE2C cells. NS = non-specific band as loading control. Western blot intensity was quantified by ImageJ and normalized to the non-specific band.

miRNAs are short ~22 nucleotide noncoding RNAs that are sequentially processed from primary transcripts (pri-miRNA) by Drosha in the nucleus and then by Dicer in the cytoplasm, thereby complexing with an RNA induced silencing complex (RISC) to modulate the stability and/or translation of their mRNA targets [50,51]. miRNAs are predicted to target at least 50% of mammalian protein-coding genes, indicating that they are involved in the regulation of a variety of cellular processes. miRNAs are widely deregulated in cancer and other diseases [50,51]. One genetic feature of neuroblastoma is loss of heterozygosity (LOH) of chromosome 1p36 or 11q23, which bears miR-34a and miR-34b/c, respectively. miR-34 is well known to possess anti-tumor functions by antagonizing oncogenes [52]. As miRNA is usually wired into a network and regulates multiple genes that are interconnected within pathways or cellular processes, we surmised that the identification of a gene network of cancer-associated miRNAs may provide an opportunity to design combined therapy by targeting the interconnected molecules. We therefore started with identification of the miR-34-targeted gene network in neuroblastoma cells. First, we verified that miR-34 was tumor suppressive in neuroblastoma cells by transfecting the three individual miR-34s into BE2C cells, a commonly used neuroblastoma cell line with *MYCN* amplification. miR-34 expression resulted in dramatic cellular differentiation, as evidenced by neurite outgrowth (Figure 1A,B). These results were consistent with previous studies showing that miR-34a has antitumor activity [53,54]. We further showed that lentiviral-mediated overexpression of miR-34b significantly reduced tumor burden in CB17 SCID mice (Figure 1C), which validated the tumor suppressive functions of miR-34 paralogs. The cellular phenotype induced by miR-34 was specific as anti-miR-34 substantially rescued the miR-34-induced differentiation and cell survival (Figure 1B). Then, we performed global gene expression profile analysis after transfecting miR-34 into BE2C cells. The three miR-34s induced expression changes (>2-fold) in numerous genes (Table S1). Although a substantial number of different genes were specifically induced by individual miRNAs, the majority of targets were commonly affected by the three miR-34s (Table S2). Gene set enrichment analysis revealed that the genes downregulated by miR-34 were most significantly enriched with miR-34 targets (Figure 1D), demonstrating the transfected miR34 hit targets in cells. Next, we used the genes commonly downregulated by miR-34s as a gene signature for a molecular concept analysis to mine the functional association between miR-34 targets and other gene signatures and pathways (including literature-based concepts, genetic perturbation, molecular mutation, clinical outcome, disease recurrence and metastasis, pathological subtypes). We found that genes affected by miR-34 were significantly associated with signatures of advanced neuroblastoma, p53 mutation, 1p36 LOH in neuroblastoma, poor clinical outcome, recurrent disease and metastasis, and target genes of PRC2 (EZH2, SUZ12) components (Figure 1E), suggesting that miR-34 is engaged in regulating a variety of pathways including EZH2 target genes. Anti-miR-34 rescued the expression of PRC2 components (Figure 1F), further validating the specificity of miR-34 in regulating PRC2 complex.

### 2.2. miR-34 Targets a 27-Gene Network Centered by EZH2

**Figure 2:**
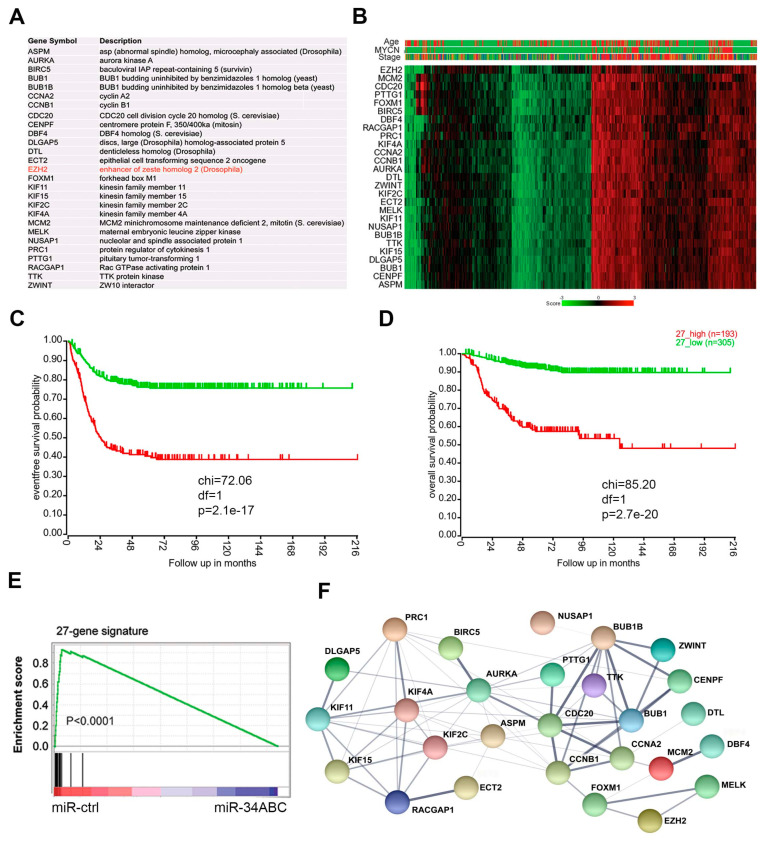
The EZH2 correlated 27-gene signature is targets of miR-34 and associated with poor outcomes of neuroblastoma. (**A**) The 27 genes whose expression are significantly correlated with EZH2 across multiple solid cancers. (**B**) Heatmap shows the 27 genes in neuroblastoma dataset (GSE45547) that contained 649 samples. Age (red  >  18 months; green  <  18 months); MYCN (red  =  amplification; green  =  non-amplification); stage (red  =  stage 4; blue  =  stage 4S; brown  =  stage 3; dark green  =  stage 2, green  =  stage 1). Scale bar indicates the expression score. (**C**) Kaplan–Meier analysis shows that the 27-gene signature is associated with poor event-free outcome in patients with neuroblastoma from dataset (GSE62564) that contained 498 samples. (**D**) Kaplan–Meier analysis shows that the 27-gene signature is associated with overall survival in patients with neuroblastoma from dataset (GSE62564) that contained 498 samples. (**E**) Gene set–enrichment analysis shows that miR-34 targets the 27-gene signature in neuroblastoma cells. (**F**) Protein–protein interaction network of the 27-gene signature encoding proteins analyzed by STRING program.

miRNA targets are often interconnected within a gene expression network. Genes co-expressed in tumors may also be associated within a signaling pathway or linked together as a module to respond to common genetic or epigenetic alterations. Given these assumptions, the analysis of genes co-expressed with *EZH2* that are also targeted by miR-34 can not only help identify novel pathways or molecular networks but also provide therapeutic targets for rational combination therapy. We generated EZH2 signatures (genes co-expressed with *EZH2* with a *R* > 0.6, *p* < 0.001, by Spearman correlation analysis) from various cancers (neuroblastoma, glioblastoma, breast cancer, colon cancer, lung cancer, and ovarian cancer) and then generated a common EZH2 co-expression signature composed of 27 genes that is shared by all these cancers (Figure 2A). Gene annotation revealed that components of the 27-gene signature are mainly involved in the cell cycle and cytokinesis (Table S3), wherein they promote cell proliferation and prevent apoptosis. The cBioPortal for Cancer Genomics database showed that the 27-gene signature is widely deregulated in various cancers (Figure S1). For example, *ASPM*, *ECT2*, *AURKA*, *BIRC5*, *CENPF,* and *EZH2* are amplified in several cancers (Figure S1). Then, we validated the presence of the 27-gene signature in a large cohort of neuroblastoma dataset [55]. In neuroblastoma, the 27 genes were highly correlated and expressed at much higher levels in patients with advanced disease, with *MYCN*-amplified tumors, and of older age (>18 months) (Figure 2B), all of which are all predictors of poor outcome [8]. In addition, high expression of the 27-gene signature in neuroblastoma was associated with worse event-free survival (Figure 2C) and overall survival (Figure 2D). It is worth to pointing out that *BIRC5*, encoding the SURVIVIN protein, is located on chromosome 17q25 that is frequently gained in high-risk neuroblastomas [1]. GSEA analysis demonstrated that the 27 genes were significantly downregulated by miR-34 (Figure 2E), indicating that this signature might function as a module or network that is antagonized by the tumor suppressor miR-34.

The finding that the 27 genes are co-expressed in various solid tumors and targeted by miR-34 indicates the biological connections of EZH2 with these genes. Positive correlations among genes usually reflect pairwise interactions between proteins [56]. Thus, enrichment for protein–protein interactions within the 27-gene signature could provide an independent line of validation for the shared function of miR-34 targets at the protein level. We analyzed the physical interaction subnetwork of the proteins encoded by the 27-gene signature by using STRING (a program for protein–protein interaction networks functional enrichment analysis). Indeed, the 27 proteins were interconnected either directly or indirectly through interacting partners and formed an interaction network (Figure 2F). These findings indicate that the EZH2 protein is functionally associated with or forms protein complexes with some of the remaining 26 proteins. 

### 2.3. Pharmacologic Inhibition of EZH2 and BIRC5 Induces a Remarkable Synergistic Effect

**Figure 3:**
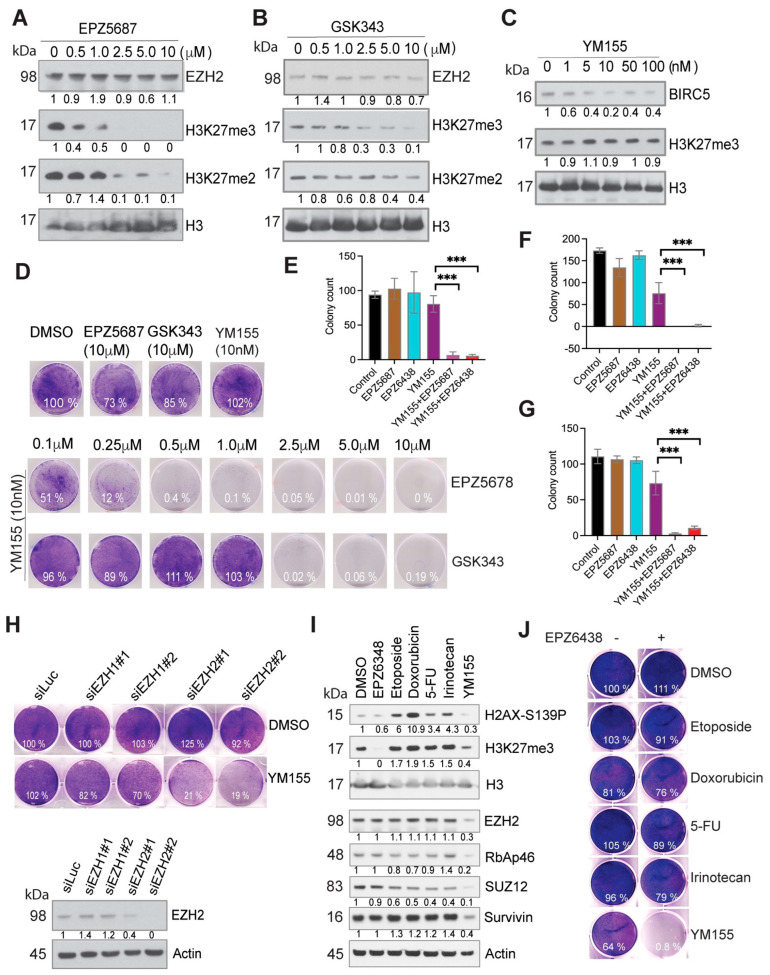
Synthetic lethality induced by EZH2 inhibitors and YM155. (**A**–**C**) Western blotting assessment of the markers with indicated antibodies. BE2C cells were treated with EZH2 inhibitor EPZ5687 (**A**), GSK343 (**B**) or BIRC5 inhibitor YM155 (**C**) with indicated concentrations for 24 h. (**D**) EZH2 inhibitors EPZ5687 or GSK343 at different concentrations in combination with 10 nM of YM155 for treatment of BE2C cells for 3 days. Cells were stained with crystal violet. (**E**–**G**) Colony quantification for BE2C (**E**), KELLY (**F**) and SKNDZ (**G**) cells treated with 10nM of YM155 (crystal violet staining in Figure S2F), and/or 5 μM of EPZ5687, 2.5 μM of EPZ6438 for 48 h, then replenished fresh media for additional 7 days. Unpaired t test is used to compare YM155 with the combinations. *** *p* < 0.01. (**H**) After siRNA knockdown of EZH1 and EZH2 in BE2C cells, Western blotting was used to assess the EZH2 expression (bottom panel). Crystal violet was used to stain the cells after siRNA knockdown of EZH1 or EZH2 followed by 3 days’ treatment of 10 nM of YM155. (**I**) Western blot for indicated markers after 24 h treatment of EPZ6438 (5 μM), Etoposide (0.5 μM), Doxorubicin (0.5 μg/mL), 5-FU (0.5 μM), Irinotecan (2.5 μM), YM155 (10 nM) (right panel). (**J**) Crystal violet was used to stain the cells after 48 h treatment with indicated agents (left panel). Colony intensity was quantified by ImageJ and normalized to DMSO control.

The 27-gene signature, as a modular network, predicts the potential functional interactions among them. The 27-gene signature is composed of multiple drug targets, whose inhibitors have been developed for cancer treatment. For example, the AURKA inhibitor MLN8237, the BIRC5 inhibitor YM155 and the MELK kinase inhibitor OTS167 have been tested in clinical trials for different types of cancers, and a TTK kinase inhibitor has also shown anti-cancer activity [57]. We adapted the synthetic lethality concept by combining specific small molecules to investigate if they can have a synergistic effect. We first treated neuroblastoma BE2C cells with the selective EZH2 inhibitor EPZ5687 or GSK343 and the BIRC5 inhibitor YM155. Western blot analysis showed that these compounds inhibited their targets. H3K27me3 was inhibited by EPZ5687 or GSK343 in a dose-dependent manner (Figure 3A,B), and BIRC5 was reduced by 2-fold by YM155 (concentration 10–100 nm; Figure 3C). As YM155 treatment did not result in a dose-dependent reduction in BIRC5, we therefore did not test a higher dose of YM155 (>100 nM) which may lead to off-target effect. Neuroblastoma cells had a modest response to YM155 (at 10 nM) after a 3-day treatment, but no cell death was observed. We also did not observe cell death at an EZH2 inhibitor concentration of 10 μM. However, the combination of EZH2 inhibitors and 10 nM of YM155 rapidly killed BE2C cells (Figure 3D) and other tested neuroblastoma cell lines NB1691 and SKNAS, and a glioma cell line SJG2 (Figure S2A–C), although each single agent had no obvious cytotoxicity. Of note, among the tested neuroblastoma cell lines, BE2C and NB1691 are *MYCN* amplified while SKNAS is not. Thus, these findings indicate that the combination of an EZH2 inhibitor and YM155 leads to pharmacological synergy. This finding was also confirmed by using a third specific EZH2 inhibitor EPZ6438 (Figure 3E–G and Figure S2D–F) and by siRNA knockdown of EZH2 (Figure 3H). EPZ6438 and EPZ5687 are similar with respect to their mechanism of action and selectivity but EPZ6438 has good oral bioavailability and is used in clinical trial now [58]. Depletion of EZH2, but not EZH1, greatly increased YM155-mediated cell death. We further verified that EZH2 inhibition specifically enhanced YM155-mediated cell killing as the combination of EPZ6438 with other commonly used chemotherapeutic agents (etoposide, doxorubicin, 5-FU, irinotecan) failed to induce synergistic effect after a 48 h treatment (Figure 3I,J). Western blot analysis showed that these drugs induced DNA damage response as indicated by phosphorylated H2AX (a DNA damage marker), but YM155 did not (Figure 3I), indicating that these DNA damaging agents hit their cellular targets at the tested concentrations. Interestingly, we noticed that 48 h treatment with YM155 reduced EZH2 expression and H3K27me3 levels (Figure 3I); however, we did not see such changes at 24 h treatment (Figure 3A,B). These data suggest that YM155 could have an inhibitory effect on EZH2 but it may not determine the synergistic effect which occurs precedingly.

Since we found the synergistic effect when neuroblastoma cells were treated with combination of YM155 with EZH2 inhibitors, we also tested other combinations of inhibitors against targets in the 27-gene signature (AURKA inhibitor MLN8237, MELK inhibitor OTS167, and TTK inhibitor AZ3146), in two different neuroblastoma cell lines BE2C (with MYCN amplification) and SKNSH (without MYCN amplification). The combination of YM155 and AZ3146 also drastically killed cancer cells (Figure S3A,B), indicating that this combination leads to pharmacological synergy. Furthermore, the effect of MELK and AURKA inhibitors was also synergistic (Figure S3A,B). Interestingly, the combination of EZH2 inhibitors with TTK inhibitor promoted cell survival (Figure S3A,B). Surprisingly, we also found that pretreatment of cells with EZH2 inhibitors partially blocked the AURKA inhibitor-mediated cell killing but not vice versa (Figure S3C). These data indicate that the components of 27-gene network have complex interactions. 

Collectively, these studies support that the 27-gene molecular network has multiple therapeutic targets. A combination of inhibitors against some of the tested targets from the 27-gene signature has a synergistic effect, thereby providing the grounds to design a novel and rational combination therapy for cancer treatment. 

### 2.4. PRC2-Mediated H3K27 Methylation Is Dispensable for the Synthetic Lethality Induced by EZH2 Inhibition and YM155

**Figure 4:**
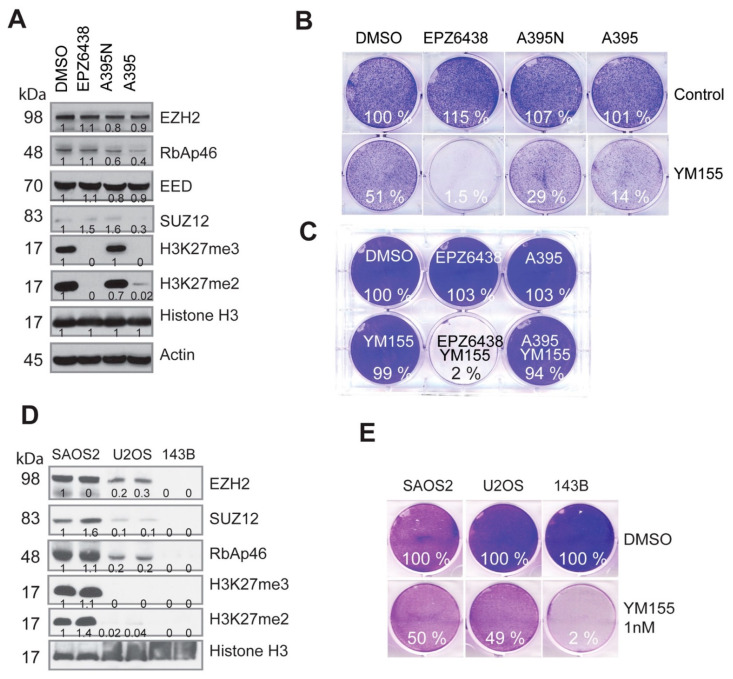
The synthetic lethality induced by EZH2 inhibitors with YM155 is independent of PRC2-mediated H3K27 methylation. (**A**) Western blot analysis for PRC2 and H3K27me3/me2 after a 48 h treatment of BE2C cells with the EZH2 inhibitor EPZ6438 (5 µM), EED inhibitor A395 (1 µM), and A395N (1 µM), the inactive analog of A395. (**B**) Cell-colony formation after 36 h treatment with EPZ6438 (5 µM), A395 (1 µM), A395N (1 µM), or YM155 (10 nM), and their combinations. (**C**) Cell-colony formation of BE2C cells after 24 h treatment from another independent experiment (EPZ6438 = 5 µM, A395 = 1 µM, YM155 = 10 nM). (**D**) Western blot analysis for PRC2 and H3K27me3/me2 in osteosarcoma cell lines. (**E**) Cell-colony formation after a 48 h treatment of U2OS cells with YM155 (1 nM) alone or in combination with EPZ6438 (5 µM). Colony intensity was quantified by ImageJ and normalized to DMSO control.

We noticed that the combination of specific EZH2 inhibitors with the BIRC5 inhibitor YM155 rapidly led to cell death (less than 24 h), which was in contrast to the slow dynamics of H3K27me3-mediated regulation of gene transcription [59]. To test if this process was specifically mediated by EZH2 or PRC2 complex, we applied a selective EED inhibitor A395 (kindly provided by SGC, Canada) to treat BE2C cells, which potently inhibited H3K27 methylation but not its inactive analog A395N (Figure 4A). Interestingly, EED inhibition failed to act synergistically with the BIRC5 inhibitor in BE2C cells at the 36 h or 24 h time point (Figure 4B,C). These findings demonstrate that PRC2-mediated H3K27 methylation does not play a critical role in EZH2-mediated synergy with YM155. We previously showed that some osteosarcoma cell lines have loss-of-function of the PRC2 complex [60]. Therefore, we took further advantage of our findings to investigate the role of PRC2. We chose three cell lines SAOS2, U2OS and 143B, which have intact, compromised, and total loss of EZH2 expression, respectively (Figure 4D). We noticed that osteosarcoma cell lines were more sensitive to YM155 than the neuroblastoma cells, we therefore chose 1 nM of YM155 for test. We hypothesized that the altered EZH2 expression may determine the response to YM155 as a monotherapy. In line with the EZH2 expression levels, SAOS2 was resistant but 143B was very sensitive to YM155 treatment (Figure 4E). H3K27me3/me2 was barely detectable in U2OS, but this cell line retained 50% of the EZH2 protein as compared with 143B, further indicating that EZH2 but not H3K27me3/me2 is a predictive marker for YM155 activity. These results indicate that in this scenario, the function of EZH2 is distinct from PRC2-mediated H3K27 methylation.

### 2.5. Combination of EZH2 Inhibitors and YM155 Induces Unfolded Protein Response and Downregulates Multiple Essential Signaling Pathways

**Figure 5:**
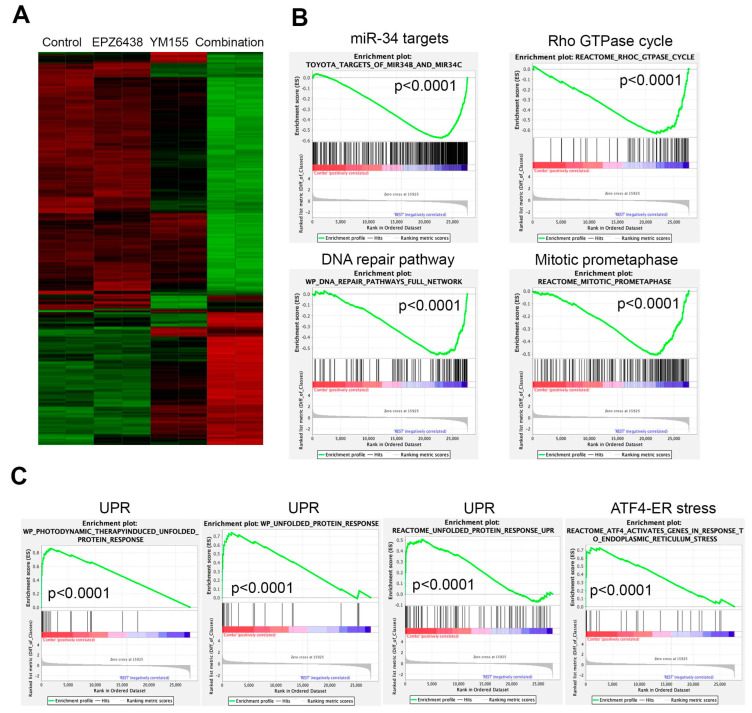
Combination of EZH2 inhibitors with YM155 perturbs multiple essential signaling pathways. (**A**) Hierarchical clustering analysis of genes altered after 12 h treatment of BE2C cells with 5 μM of EPZ6438, 5 nM of YM155, or combination. (**B**) Gene set-enrichment analysis shows gene signatures and pathways downregulated by combination of EPZ6438 and YM155 after 12 h treatment. (**C**) Gene set-enrichment analysis shows gene signatures and pathways upregulated by combination of EPZ6438 and YM155 after 12 h treatment.

To understand the signaling pathways leading to the rapid cell killing by the combination of EZH2 inhibitors with YM155, we performed global gene expression profiling analysis after BE2C cells were treated with each individual agent or combination for 12 h. Since 5 nM of YM155 induced a comparable reduction in BIRC5 to 10 nM (Figure 3C), for the purport of reducing potential off-target effect as much as possible, we chose 5 nM of YM155. The results demonstrated that the combination induced dramatic changes in gene expression, while no obvious transcriptomic alterations were observed for EZH2 inhibitor alone after 12 h treatment although YM155 induced mild changes (Figure 5A). GSEA analysis revealed that the genes downregulated by the combination treatment were significantly enriched with miR-34 targets, leading to the conclusion that these genes may be rewired to the targets of miR-34. Other top downregulated pathways by the combination were involved in Rho GTPase cycle, DNA repair and mitosis (Figure 5B), all of which are essential to cell survival. Pathways significantly upregulated by the combination were unfolded protein response (UPR) and ATF4-mediated endoplasmic reticulum stress (Figure 5C). It is well known that ATF4 is a key player of UPR and cell death [61]. These data indicate that the combination of EZH2 inhibitors with YM155 perturbed multiple signaling pathways essential to cell survival, warranting further studies in the future. 

## 3. Discussion

Combination therapy is usually required for cancer drugs to achieve higher clinical efficacy. For example, chemotherapy for neuroblastoma includes combinations of carboplatin, cyclophosphamide, doxorubicin, and etoposide. Targeted therapy based on the unique molecular features of cancer cells has been very successful for some diseases. However, designing an optimal combination of targeted therapies to obtain high efficacy remains challenging because the relationship between the 2 targets is unknown in most cases. Another concern is that one drug might hinder the effect of another drug. We surmised that simultaneous disruption of key components of the 27-gene molecular network targeted by miR-34 would result in a synergistic antiproliferative effect, which can result in an increase in therapeutic efficacy. This concept was based on the following findings: (1) The 27-gene signature centered on EZH2, identified across many types of cancers, is composed of multiple therapeutic targets such as EZH2, AURKA, MELK, TTK, KIF11, and BIRC5, whose inhibitors have been developed for cancer treatment. (2) miR-34 members, which are targets of p53 and whose function is frequently lost in cancers, target the 27-gene signature (miR-34 inhibits the expression of all 27 genes in neuroblastoma). (3) Previous studies show that a single miRNA targets multiple genes that are interconnected through pathways or cellular processes [50,51]. We therefore propose that EZH2 and the remaining 26 genes are intrinsically connected to form a network that is essential for cell viability. Indeed, the 27 gene products formed a protein–protein interaction network. (4) Moreover, gene annotation revealed that all 27 gene products are involved in the cell cycle process and are phosphoproteins. (5) We tested cell viability by using different combinations of inhibitors for network components. Although we have not tested all possible combinations, the combinations of EZH2 and BIRC5 inhibitors resulted in remarkable synergistic activity. These data strongly support our hypothesis that molecular networks can be exploited to design combination therapies.

Although it is well established that EZH2 is a key component of the PRC2 complex that silences gene expression by methylating H3K27, its role targeting non-histone substrates has been shown to be important in some cancers. One study showed that the oncogenic function of EZH2 in cells of castration-resistant prostate cancer is independent of PRC2-mediated transcriptional repression [44]. EZH2 can also methylate STAT3 to increase STAT3 activity in glioblastoma stem cells [29]. Most of the 27 gene components regulate mitosis. Several studies show that EZH2 can be phosphorylated by cyclin-dependent kinases in the mitotic phase [45,46,47], which might link to regulation of PRC2 activity or long non-coding RNA binding for gene silencing. We believed that EZH2 has a PRC2-independent function in the 27-gene signature, and the findings in this study strongly support our hypothesis: (1) Combining a BIRC5 inhibitor with an EZH2 inhibitor but not an EED inhibitor (both key components of the PRC2 complex required for H3K27 methylation) leads to a synergistic effect, despite complete inhibition of H3K27me3 by both inhibitors. (2) Cells that retain EZH2 expression but have complete loss of H3K27me3 are less sensitive to the combination of EZH2 and BIRC5 inhibitors than the cells that have a complete loss of EZH2 protein. (3) the 27-gene signature does not include other PRC2 components (e.g., SUZ12, EED, or RBAp46/48). Cancer genomic sequencing data also suggest that EZH2 might have a PRC2-independent function. For example, in malignant peripheral nerve sheath tumor, PRC2 is recurrently inactivated through component EED or SUZ12 but not EZH2, despite a complete loss of H3K27me3 [34]. Therefore, the synthetic lethality achieved by using a combination of EZH2 inhibitors in this scenario is independent of its epigenetic function, namely, PRC2-mediated methylation of lysine 27 on histone H3.

Nearly all the proteins encoded by the 27 genes are involved in the regulation of mitosis, a very dynamic process in which posttranslational modifications occur and protein complexes associate and disassociate. Interestingly, all protein products encoded by the 27 genes are phospho-proteins, and phosphorylation is known to play an important role in regulating cell cycle progression. EZH2 is phosphorylated by CDK1 in a cell cycle-dependent manner [46,62], indicating that EZH2 might work in conjunction with these proteins during cell cycle progression. *BIRC5* not only is frequently amplified at chromosome 17q in neuroblastoma [63] but also plays an important role in regulating apoptosis and the mitotic spindle checkpoint [64]. TTK (also called Mps1) is a dual-specificity kinase critical for recruiting spindle assembly checkpoint proteins to unattached kinetochores, formation of the mitotic checkpoint complex and, thus, APC/C inhibition during mitosis [65]. Therefore, the functions of EZH2 are likely involved in regulating mitosis in the 27-gene network in a H3K27me3-independent way. While the mechanism of action of YM155 is disputable [66], a recent study confirmed BIRC5 as a critical YM155 target in neuroblastoma [67]. DNA damage response induced by YM155 [66] was not observed when cells were treated with the concentrations and timing used in our study.

### Limitations of This Study

The mechanism of the synergistic combination of EZH2 inhibitors and YM155 remain to be elucidated in the future. Translating the combination therapy into clinic could be challenging as YM155 has poor pharmacokinetic properties which makes it difficult to test in preclinical models. It also warrants further studies to test whether combined EZH2 inhibition with YM155 leads to interruption of mitosis and/or DNA repair pathways. It would be necessary in the future to determine the IC_50_ for each drug for evaluating the additive or synergistic effect of the combinations used.

## 4. Materials and Methods

Cell culture and compounds. SJG2 (St Jude), SAOS2, U2OS and 143B (ATCC) cells were maintained in DMEM medium, supplemented with 10% FCS (HyClone, Thermo, Waltham, MA, USA), 1% L-glutamine (MediaTech, Hsinchu, Taiwan), 1% penicillin–streptomycin (Lonza, Basel, Switzerland). BE2C, SK-N-AS, IMR-32, KELLY, SKNDZ, and SK-N-SH (all from ATCC) and NB-1691 cells (Peter Houghton, Columbus, OH, USA) were maintained in RPMI medium supplemented with 10% FCS (HyClone, Thermo), 1% L-glutamine (MediaTech), and 1% penicillin–streptomycin (Lonza). All human-derived cell lines were validated by short tandem repeat (STR) profiling using PowerPlex^®^ 16 HS System (Promega, Madison, WI, USA) once a month. Once a month, a polymerase chain reaction (PCR)-based method was used to screen for mycoplasma employing the LookOut^®^ Mycoplasma PCR Detection Kit (MP0035, Sigma-Aldrich, St. Louis, MO, USA) and JumpStart™ Taq DNA Polymerase (D9307, Sigma-Aldrich) to ensure that cells were free of mycoplasma contamination. EZH2 inhibitors EPZ-5687, EPZ-6438, and GSK343; Aurora A kinase inhibitor MLN8237, BIRC5 inhibitor YM-155, TTK inhibitor AZ3146, and MELK inhibitor OTS167 were purchased from Selleck Chemicals. The EED inhibitor A395 and its inactive analog A395N were kindly provided by SGC, Canada.

miRNA and siRNA. The Pre-miR™ miRNA Precursor molecules (hsa-miR-34a, hsa-miR-34b, hsa-miR-34c, and the control) are small, chemically modified double-stranded RNA molecules designed to mimic endogenous mature miRNAs, which were purchased from Ambion (Life Technologies, Carlsbad, CA, USA). The anti-miR-34s were purchased from Life Technologies. siRNA oligos for knockdown of EZH1 (#1, 5-GGAAAGGUCUAUGACAAAU-3; #2, 5-GAAAGCGACAUGCUAUUGA-3), EZH2 (#1, 5-GAGGACGGCUUCCCAAUAA-3; #2, 5-GCUGAAGCCUCAAUGUUUA-3), and siRNA luciferase control (5-CTTACGCTGAGTACTTCGA-3) were synthesized by Dharmacon, Thermo Scientific (Waltham, MA, USA). miRNA or siRNA were reverse transfected using RNAiMAX (Invitrogen, Waltham, MA, USA) according to the manufacture’s protocol. Lenti-miR-34b and control were purchased from Abmgood company.

Western blotting. Cell lysates were either prepared using RIPA buffer (Cell Signaling, Danvers, MA, USA) supplemented with protease inhibitors (Roche complete mini), followed by 5 min heating at 75 °C after addition of 2XSample Loading buffer (100 mM Tris-HCl pH 6.8, 4% SDS, 20% Glycerol, 5% β- mercaptoethanol, 0.2% Bromophenol blue). Alternatively, the cells were directly lysed in 2XSample Loading buffer followed by 20 min heating at 95 °C. The samples were separated on 4–12% Tris- glycine SDS-PAGE gels (Invitrogen) and transferred to PVDF membrane (Millipore, Burlington, MA, USA). Membranes were blocked for 1 h in TBS buffer with 5% milk and 0.1% Tween 20, followed by overnight incubation with primary antibodies at 4 °C. Membranes were washed for 3 × 5 min at room temperature in TBS-T buffer. Mouse and rabbit HRP-conjugated secondary antibodies (ThermoFisher, 1:5000) were incubated for 1 h at room temperature followed by washing 3 × 5 min at room temperature in TBS-T. For detection, membranes were exposed to Pierce ECL Western Blotting Substrate and detected on Hyblot CL film (Denville Scientific Inc., Holliston, MA, USA). All the Western blot antibodies are listed: Rabbit anti-EZH2 (D2C9) Antibody (Cell Signaling Technology, Cat#5246, RRID:AB_10694683), Rabbit anti-SUZ12 (D39F6) Antibody (Cell Signaling Technology, Cat#3737, RRID:AB_2196850), Rabbit anti-RbAp46 (D39F6) Antibody (Cell Signaling Technology Cat# 4633, RRID:AB_1904116), Rabbit anti-H3K27me3 Antibody (Cell Signaling Technology Cat# 9733, RRID:AB_2616029), Rabbit anti-H3K27me2 Antibody (Cell Signaling Technology Cat# 9728, RRID:AB_1281338), Rabbit anti-H3 Antibody (Cell Signaling Technology Cat# 4499, RRID:AB_10544537), Rabbit anti-BIRC5 Antibody (Cell Signaling Technology Cat# 2808, RRID:AB_2063948), Mouse anti-phospho-Histone H2A.X (Ser139) Antibody, clone JBW301 (Millipore Cat# 05-636, RRID:AB_309864), Mouse anti-EED Antibody, clone AA19 Millipore (Cat# 09-774, RRID:AB_1587000), Mouse Anti-beta-Actin Monoclonal Antibody, and Clone AC-15 (Sigma-Aldrich, Cat# A5441, RRID:AB_476744). 

Crystal Violet Staining. For short-term assessment of drug combination effect, 0.8 × 10^5^ of cells were seeded in 6-well plate in singlicate. Next day, cells were treated with YM155 and EZH2 inhibitors for 24–48 h. After removing media, cells were washed with Dulbecco’s phosphate-buffered saline without calcium or magnesium (DPBS, Lonza) and treated with 4% formaldehyde in PBS (PFA) for 20 min. Once PFA was removed, cells were stained with 0.1% crystal violet stain for 1 h. For long-term assessment of drug combination effect, 3000 cells of each neuroblastoma cell line (BE2C, KELLY, SKNDZ) seeded in 6-well plate in triplicates, treated with drugs for 48 h, then replenished with fresh media without drugs for additional 7 days, and then stained with crystal violet for colony counting.

Affymetrix Microarray Analysis. miRNA was transfected into BE2C cells. 72 h later, RNA was extracted using the RNeasy kit from QIAGEN, and was subjected to hybridization using an Affymetrix HT HG-U133+ PM 16-Array Plate done by the St Jude Hartwell Center. For drug treatments with EPZ6438, YPM155 or combination, BE2C cells were harvested for RNA extraction after 12 h treatment, which was subject to hybridization using an Affymetrix HUGENE2.0 Plate done by the St Jude Hartwell Center Differential gene expression was analyzed using the GenePattern program (http://www.broadinstitute.org/cancer/software/genepattern/, accessed on 15 September 2022) while the gene signature was analyzed using the Gene Set Enrichment Analysis (GSEA) program (http://www.broadinstitute.org/gsea/index.jsp, accessed on 15 September 2022). Cluster analysis was performed by Spotfire program.

Identification of 27-gene signature. To identify the genes that are significantly co-expressed with *EZH2*, we queried EZH2 with R2: Genomics Analysis and Visualization Platform (http://hgserver1.amc.nl/cgi-bin/r2/main.cgi, accessed on 15 September 2022) by searching EZH2 correlated genes in multiple datasets including EXPO breast, EXPO lung, EXPO colon, Bowtell ovarian, Versteeg neuroblastoma, TCGA glioblastoma. We then define the most significant correlated genes (Pearman correlation *R* > 6 or 7, *p* < 1 × 10^−10^) as the co-expression signatures. Then, we chose the 27 common genes among these signatures as the signature for survival analysis and functional studies. The heatmap for differential expression of 27 genes in high-stage and low stage samples was generated using the Kocak dataset (GSE455547). The K-means method was applied to stratify the 27 gene signature for event-free survival and overall survival using the SEQC dataset (GSE49710).

Oncomine concept analysis of the miR-34 signature. We used 481 genes downregulated by miR-34s as the query signature. We imported these gene lists into Oncomine as custom concepts. We then nominated significantly associated cancer concepts with odds ratio > 2.0 and *p* < 1 × 10^−6^. We exported these results as the nodes and edges of a concept association network and visualized the network using Cytoscape version 2.8.2.

Pathway network analysis. The 27 genes were uploaded into STRING program (https://string-db.org, accessed on 15 September 2022) for network interaction analysis with confidence threshold 0.4.

Animal Experiments. All murine experiments were done in accordance with a protocol approved by the Institutional Animal Care and Use Committee of St. Jude Children’s Research Hospital. Lentivirus was used to transduce BE2C cells. After transduction, 1 μg/mL of puromycin was used for selection of stable knockdown. Subcutaneous xenografts were established in male CB-17 severe combined immunodeficient mice with age of 4–6 weeks (Taconic, Hudson, NY, USA), in a random manner. Tumors measurements were done weekly using handheld calipers, and volumes calculated as width π/6 × d3 where d is the mean of two diameters taken at right angles. The tumor implantation and volume measurement were performed by Core facility and thus blinded to authors but not to the Core facility staff.

## 5. Statistical Analysis

Student *t* test was used in this study. *p* < 0.05 and <0.01 were considered statistically significant and highly significant, respectively.

Microarray data are deposited at the Gene Expression Omnibus under accession number GSE49845, GSE208593.

## 6. Conclusions

In conclusion, we identified a 27-gene signature centered on EZH2 that led to the development of combination therapies. Combination of the BIRC5 inhibitor YM155 and EZH2 inhibitors achieved remarkable synergistic effect in cancer cell killing and this is independent of the canonical functions of EZH2 in methylating H3K27.

## Data Availability

Microarray data are deposited at the Gene Expression Omnibus under accession number GSE49845, GSE208593. Part of the data presented in this study are openly available in database, as discussed in the Material and Methods section.

## Supplementary Materials

The following supporting information can be downloaded at https://www.mdpi.com/article/10.3390/cancers15010208/s1, Figure S1: Genetic alterations of 27-gene signature; Figure S2: Synergistic effect of EZH2 in combination with YM155; Figure S3: Synergistic effect of YM155 in combination with other inhibitors against targets in 27-gene signature. Table S1: Genes downregulated and upregulated by miR34s; Table S2: Common genes downregulated and upregulated by miR34s; Table S3: 27-gene annotation.
