# Supplementary material for: Remarkable Synergy When Combining EZH2 Inhibitors with YM155 Is H3K27me3-Independent
Source: Cancers (Basel). 2022 Dec 29;15(1):208. doi: 10.3390/cancers15010208 (PMC9818370; doi:10.3390/cancers15010208)
Supplement: Supplementary file 1 [file cancers-15-00208-s001.zip › cancers-1945850-SI.pdf]

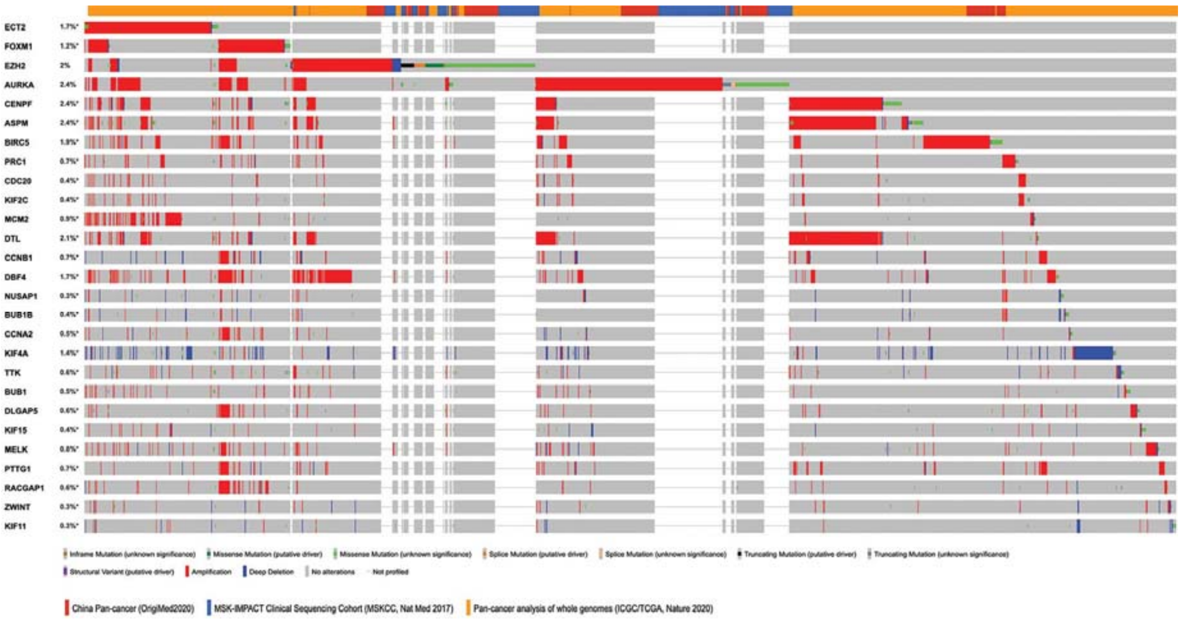

Figure S1. Dysregulation of 27-gene across many types of cancer.

The 27 genes were uploaded into cBioportal program (https://www.cbioportal.org/) and the genetic alterations were analyzed using the datasets including Pan-cancer analysis of whole genomes (ICGC/TCGA, Nature 2020, containing 2922 samples), MSK-IMPACT Clinical Sequencing Cohort (MSKCC, Nat Med 2017, containing 10945 samples), China Pan-Cancer (OrigiMed2020, containing 10194 samples).

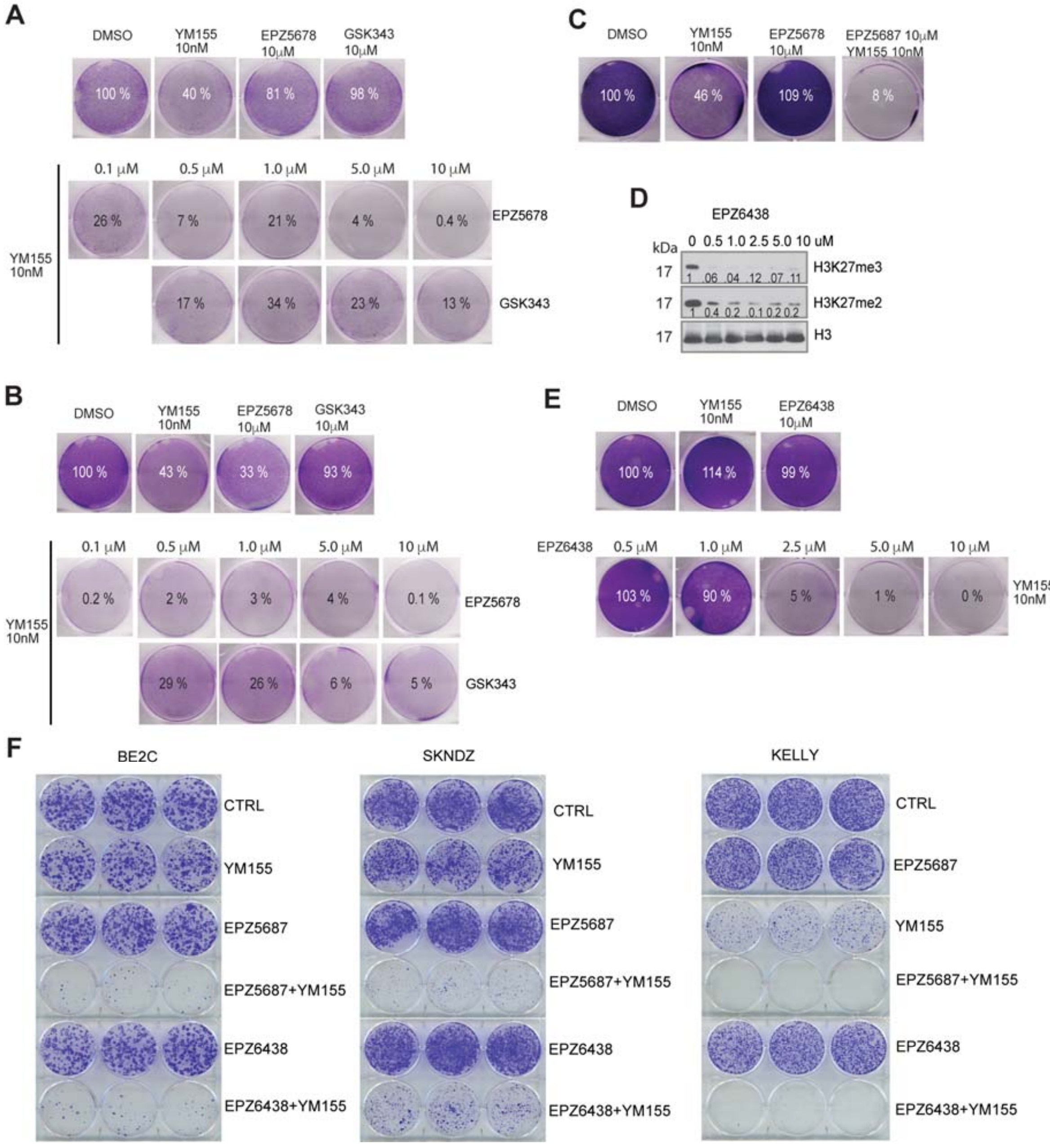

Figure S2. Synergistic effect induced by EZH2 inhibitors and YM155. (A, B) EZH2 inhibitors EPZ5687 or GSK343 at different concentrations in combination with 10 nM of YM155 for treatment of NB1691 and SKNAS cells for 3 days. Cells were stained with crystal violet. (C) EZH2 inhibitors EPZ5687 at 10μM in combination with 10 nM of YM155 for treatment of SJG2 cells for 3 days. Cells

were stained with crystal violet. (D) Western blotting assessment of the markers with indicated antibodies. BE2C cells were treated with EZH2 inhibitor EPZ6438 (with indicated concentrations. (E) EZH2 inhibitors EPZ6438 at different concentrations in combination with 10 nM of YM155 for treatment of BE2C cells for 3 days. Cells were stained with crystal violet. Colony intensity was quantified by ImageJ and normalized to DMSO control. (F) Colony forming assay for BE2C (E), KELLY (F) and SKNDZ (G) cells treated with 10nM of YM155, and/or 5mM of EPZ5687, 2.5mM of EPZ6438 for 48 hours, then replenished fresh media for additional 7 days.

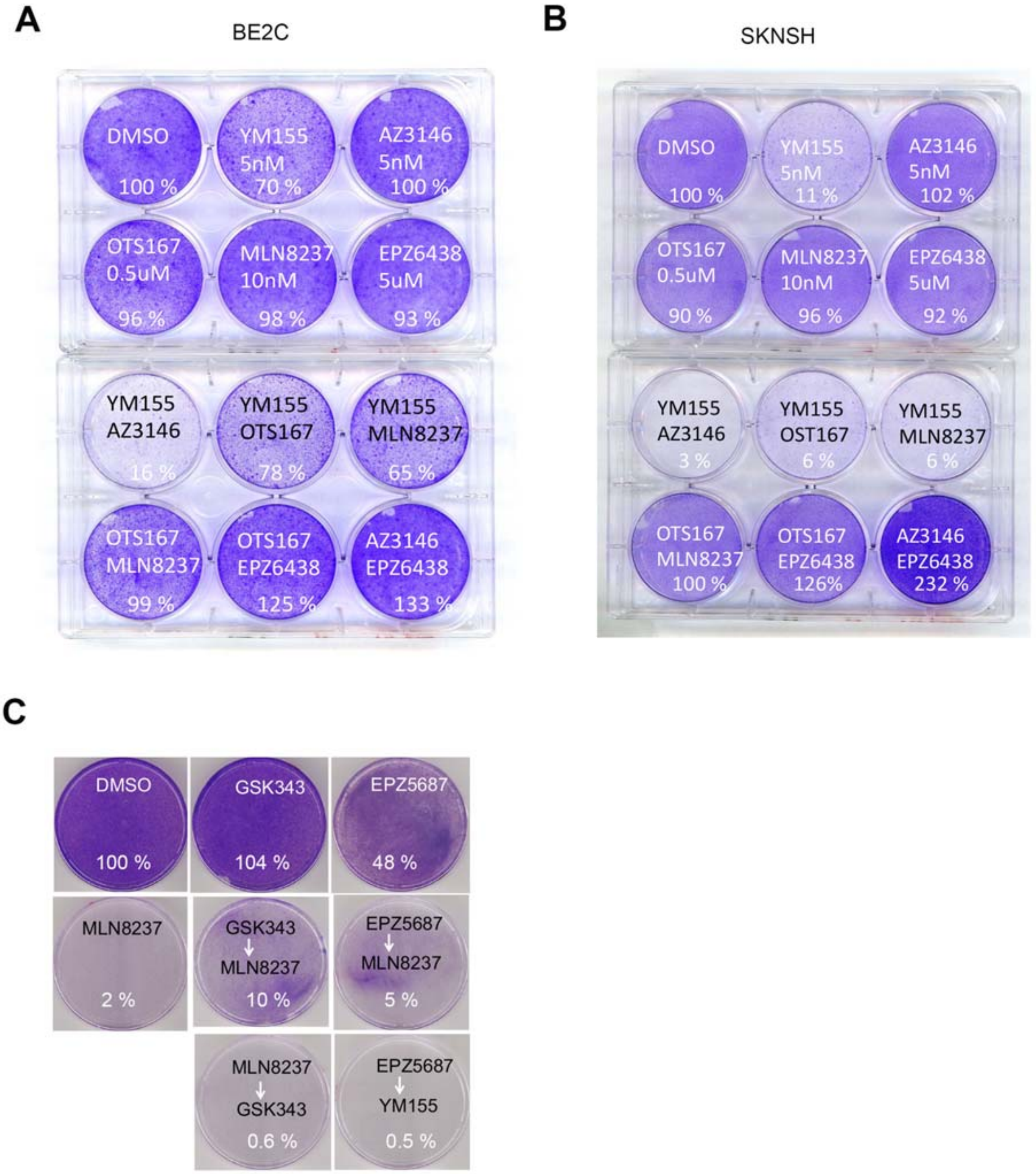

Figure S3. Combinations of AZ3146 and YM155 induced substantial synergy to kill cancer cells. (A, B) Different combinations with indicated concentrations for treatment of BE2C and SKNSH cells for 2 days. Cells were stained with crystal violet. (C) Cells were pretreated with indicated drugs for 24

hours, followed by second drug administration. Cells were stained with crystal violet. Colony intensity was quantified by ImageJ and normalized to DMSO control.
