# Supplementary material for: Remarkable Synergy When Combining EZH2 Inhibitors with YM155 Is H3K27me3-Independent
Source: Cancers (Basel). 2022 Dec 29;15(1):208. doi: 10.3390/cancers15010208 (PMC9818370; doi:10.3390/cancers15010208)
Supplement: Supplementary file 1 [file cancers-15-00208-s001.zip › cancers-1945850-supplementary.pdf]

Figure 1F

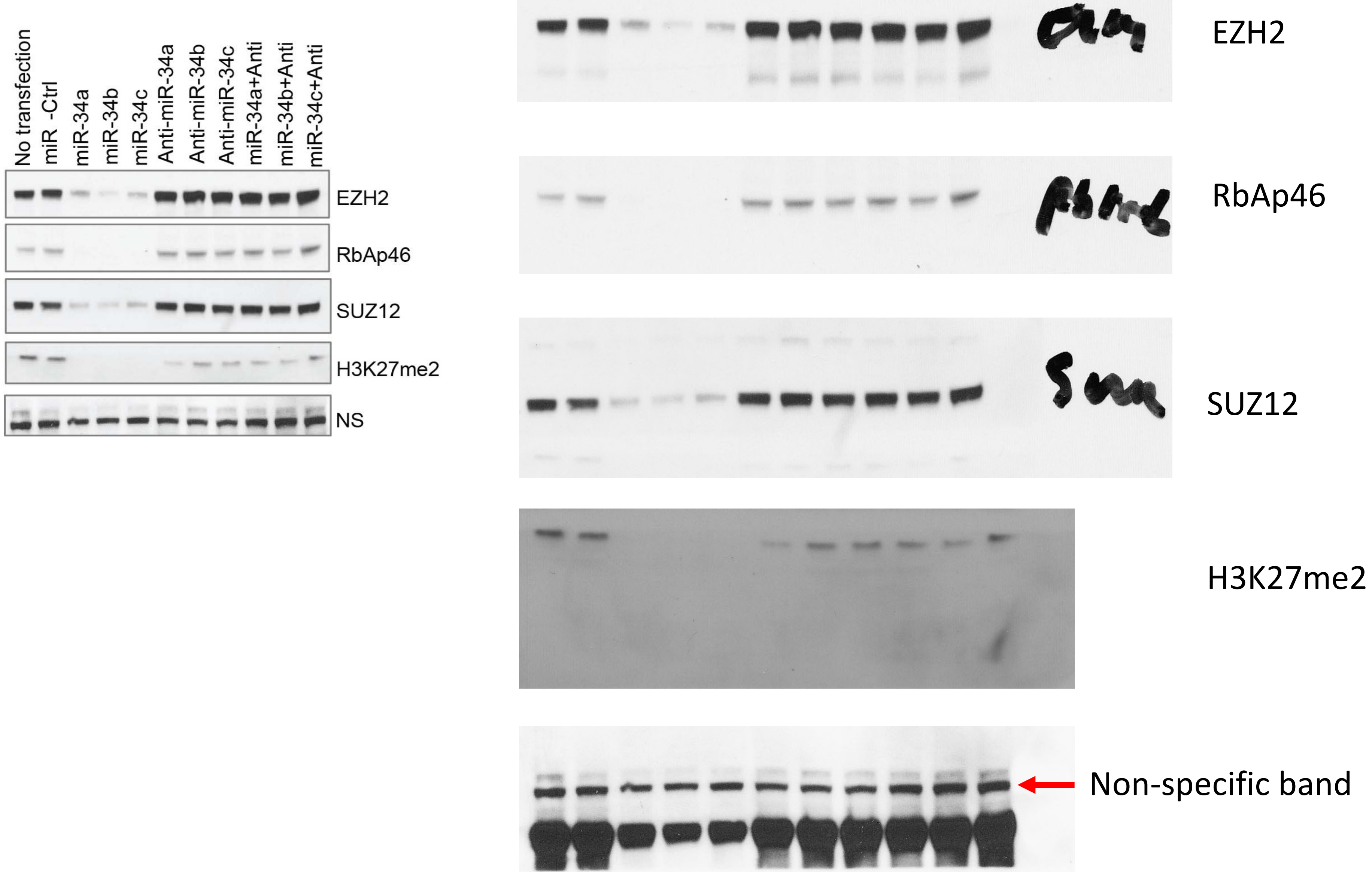
No transfection
miR -Ctrl
miR-34a
miR-34b
miR-34c
Anti-miR-34a
Anti-miR-34b
Anti-miR-34c
miR-34a+Anti
miR-34b+Anti
miR-34c+Anti
EZH2
RbAp46
SUZ12
H3K27me2
NS
EZH2
RbAp46
SUZ12
H3K27me2
Non-specific band

Figure 3A

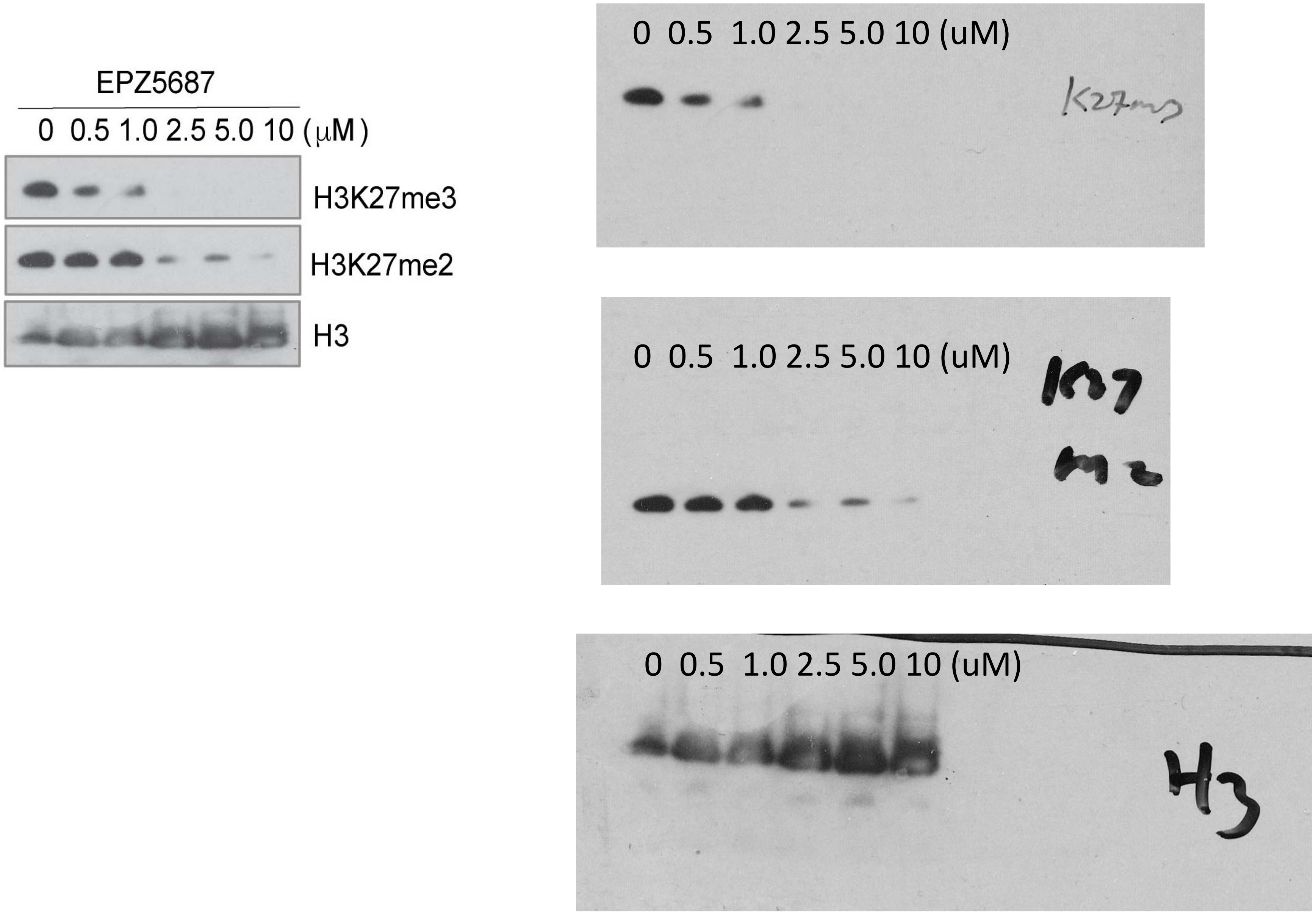
EPZ5687
0 0.5 1.0 2.5 5.0 10 (μM)
H3K27me3
H3K27me2
H3
0 0.5 1.0 2.5 5.0 10 (uM)
0 0.5 1.0 2.5 5.0 10 (uM)
0 0.5 1.0 2.5 5.0 10 (uM)
H3

Figure 3B

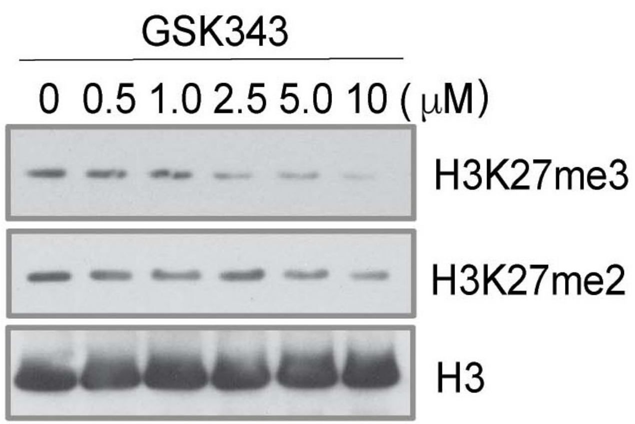

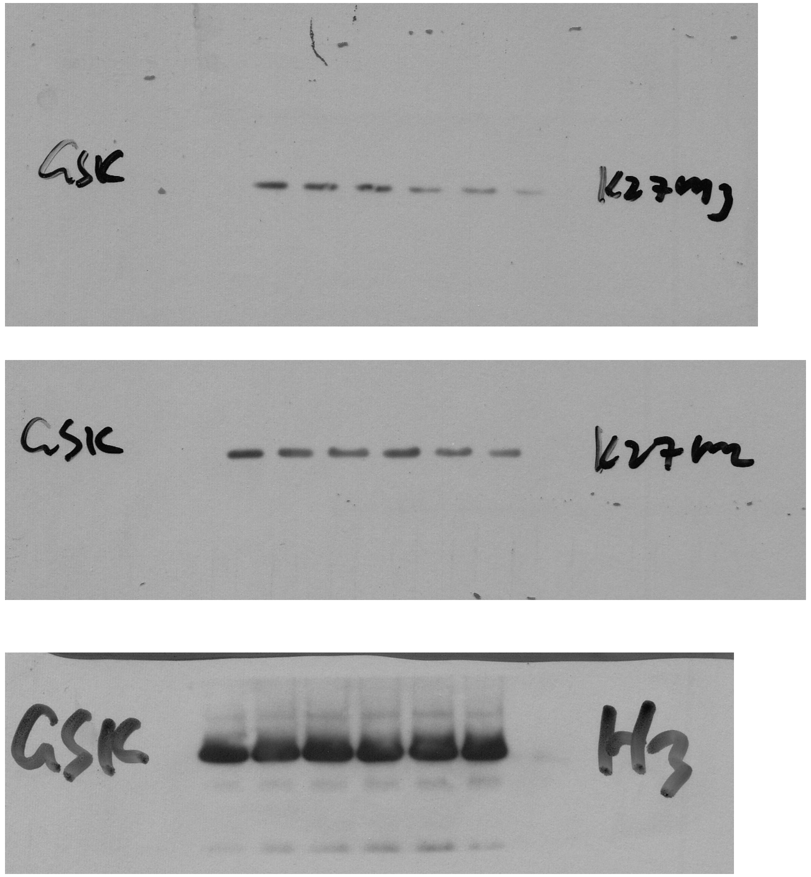

Figure 3C

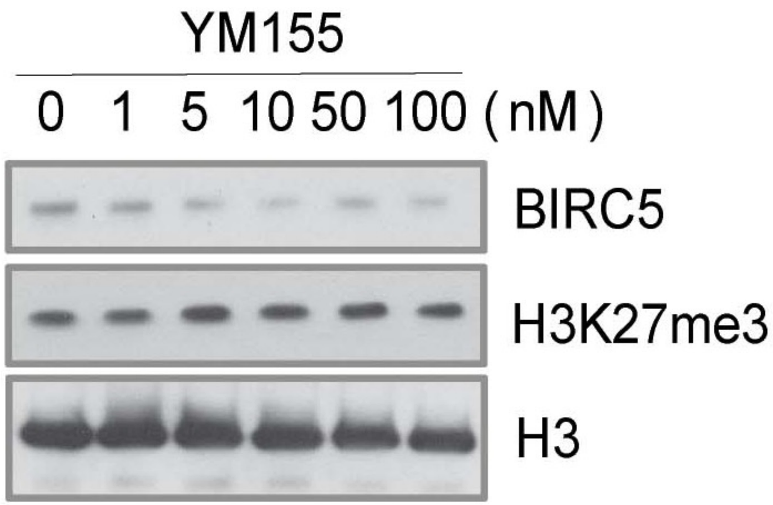

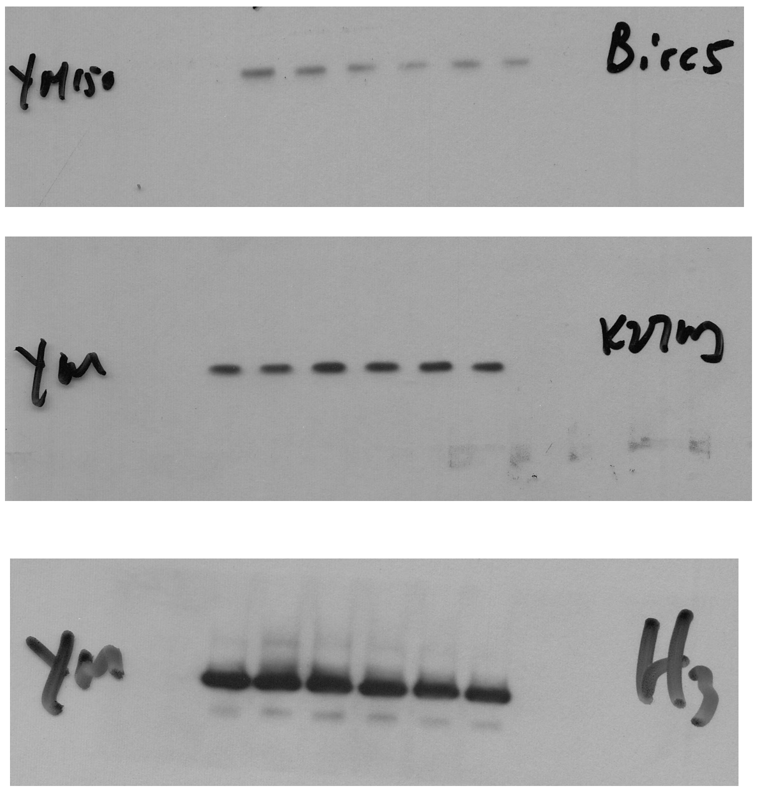

Figure 3E

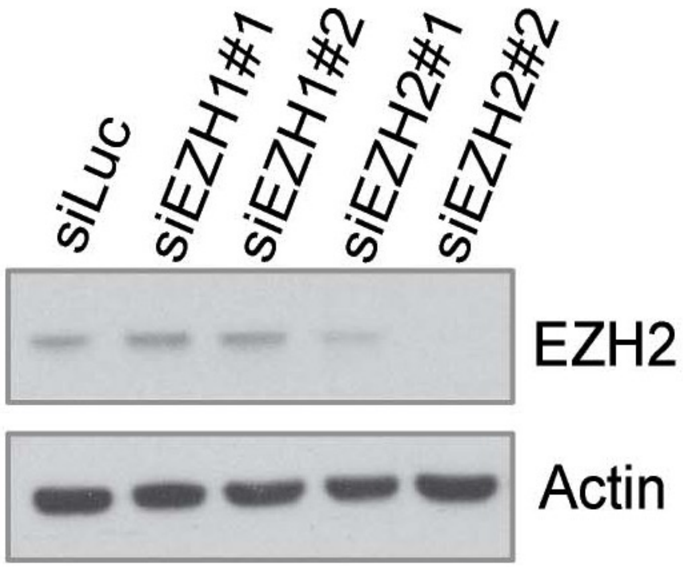
siLuc
siEZH1#1
siEZH1#2
siEZH2#1
siEZH2#2
EZH2
Actin

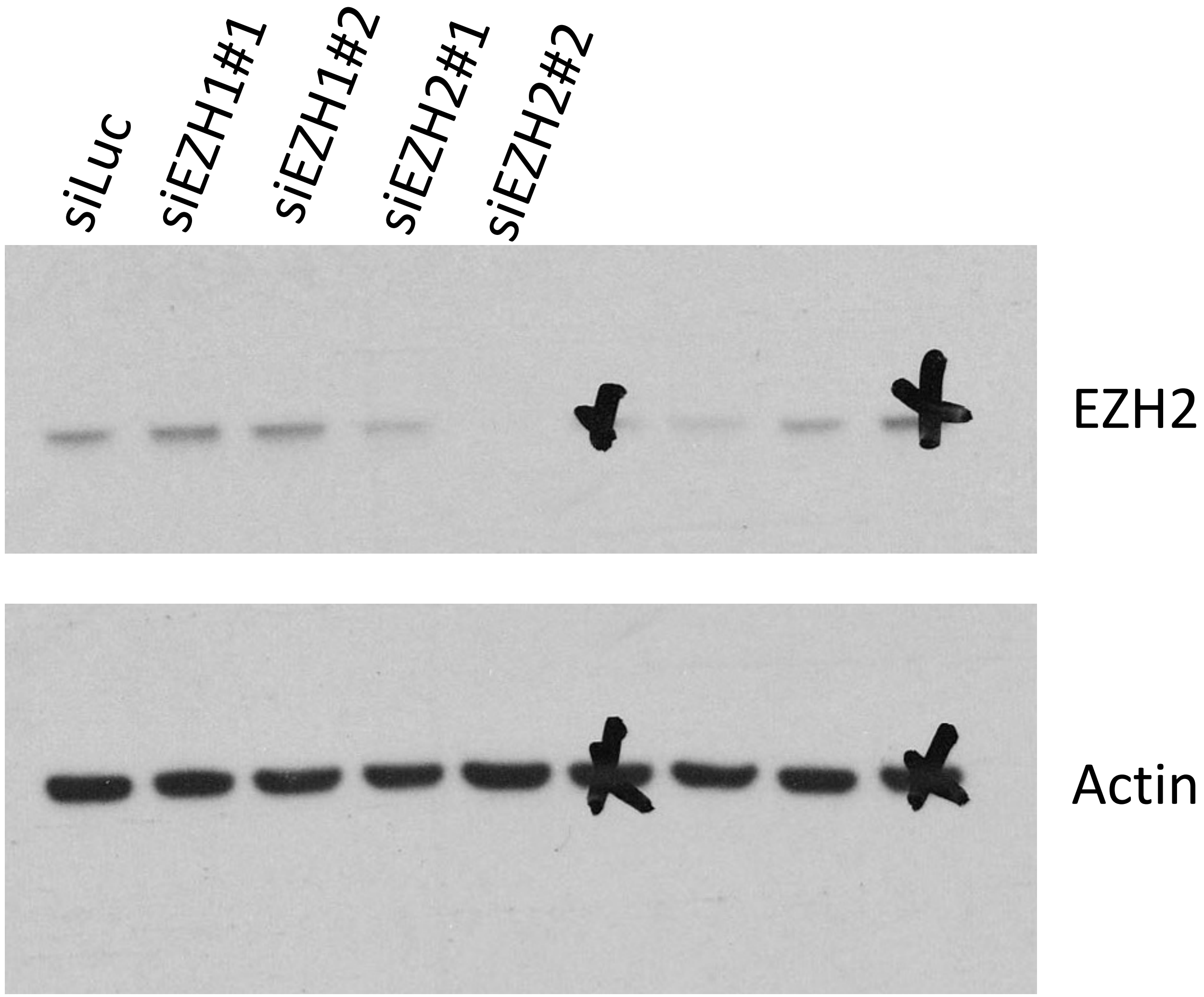
siLuc
siEZH1#1
siEZH1#2
siEZH2#1
siEZH2#2
EZH2
Actin

Figure 3F

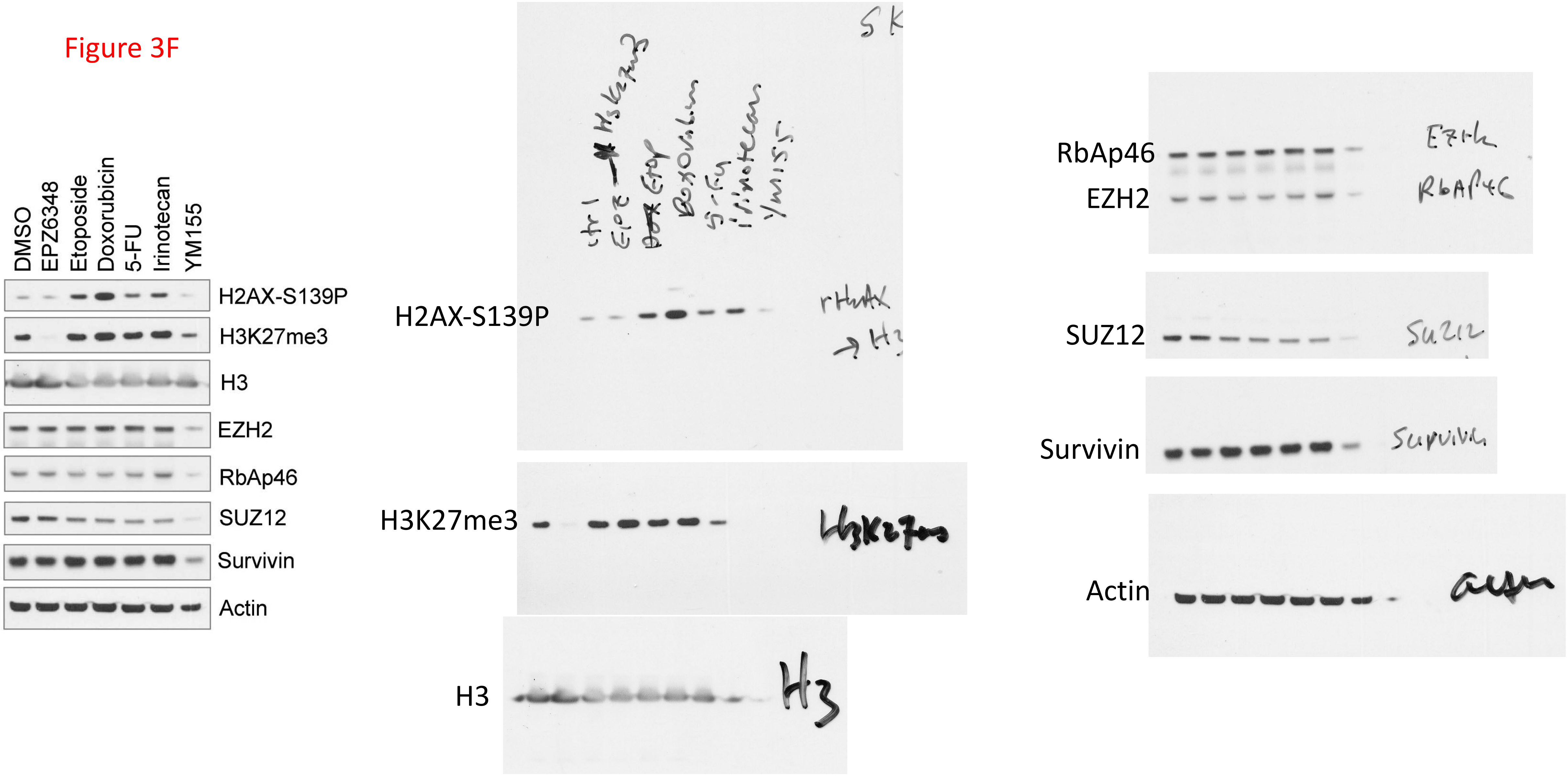
DMSO
EPZ6348
Etoposide
Doxorubicin
5-FU
Irinotecan
YM155
H2AX-S139P
H3K27me3
H3
EZH2
RbAp46
SUZ12
Survivin
Actin
H2AX-S139P
H3K27me3
H3
RbAp46
EZH2
SUZ12
Survivin
Actin

Figure 4A

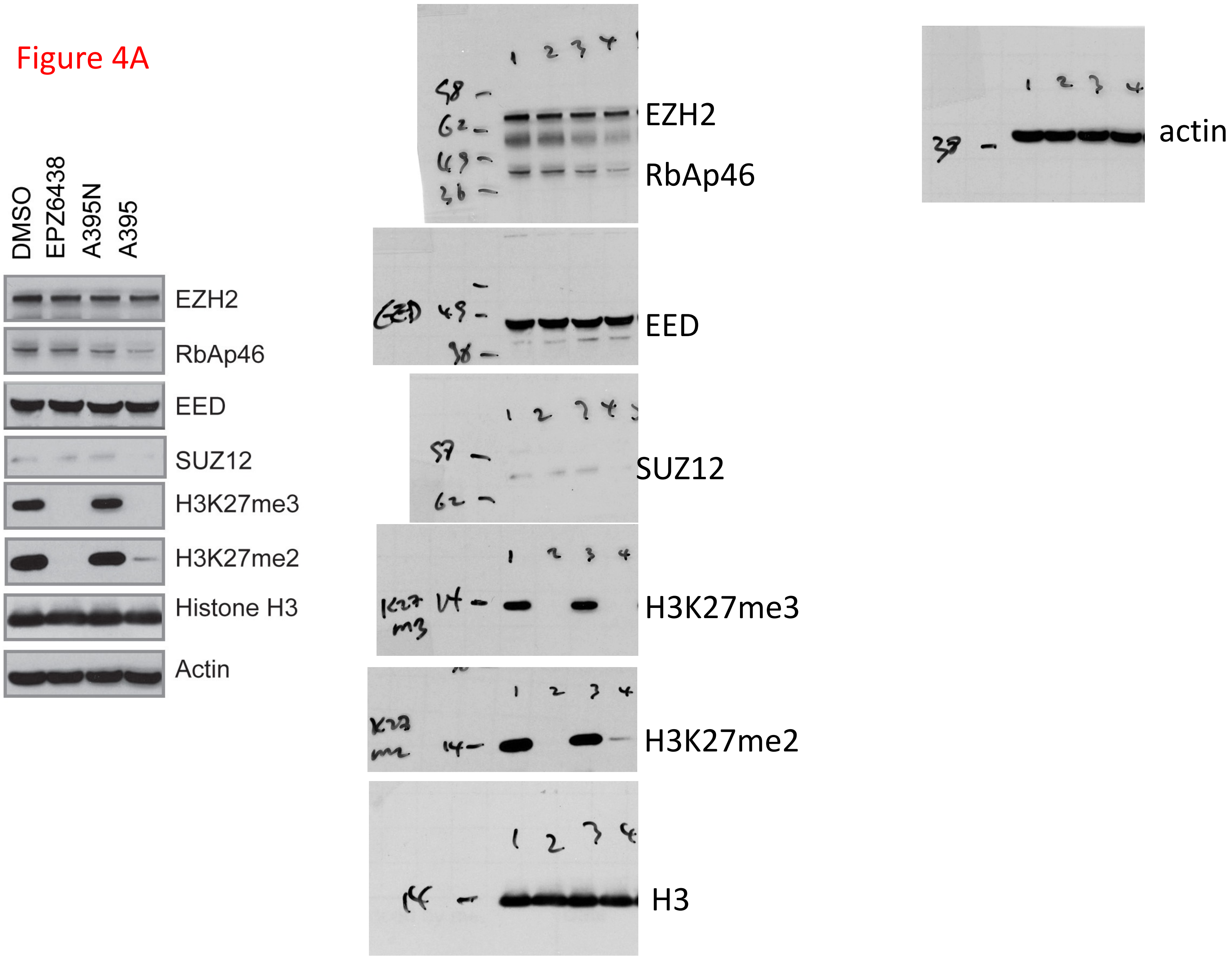
DMSO
EPZ6438
A395N
A395
EZH2
RbAp46
EED
SUZ12
H3K27me3
H3K27me2
Histone H3
Actin
EZH2
RbAp46
EED
SUZ12
H3K27me3
H3K27me2
H3
actin

Figure 4D

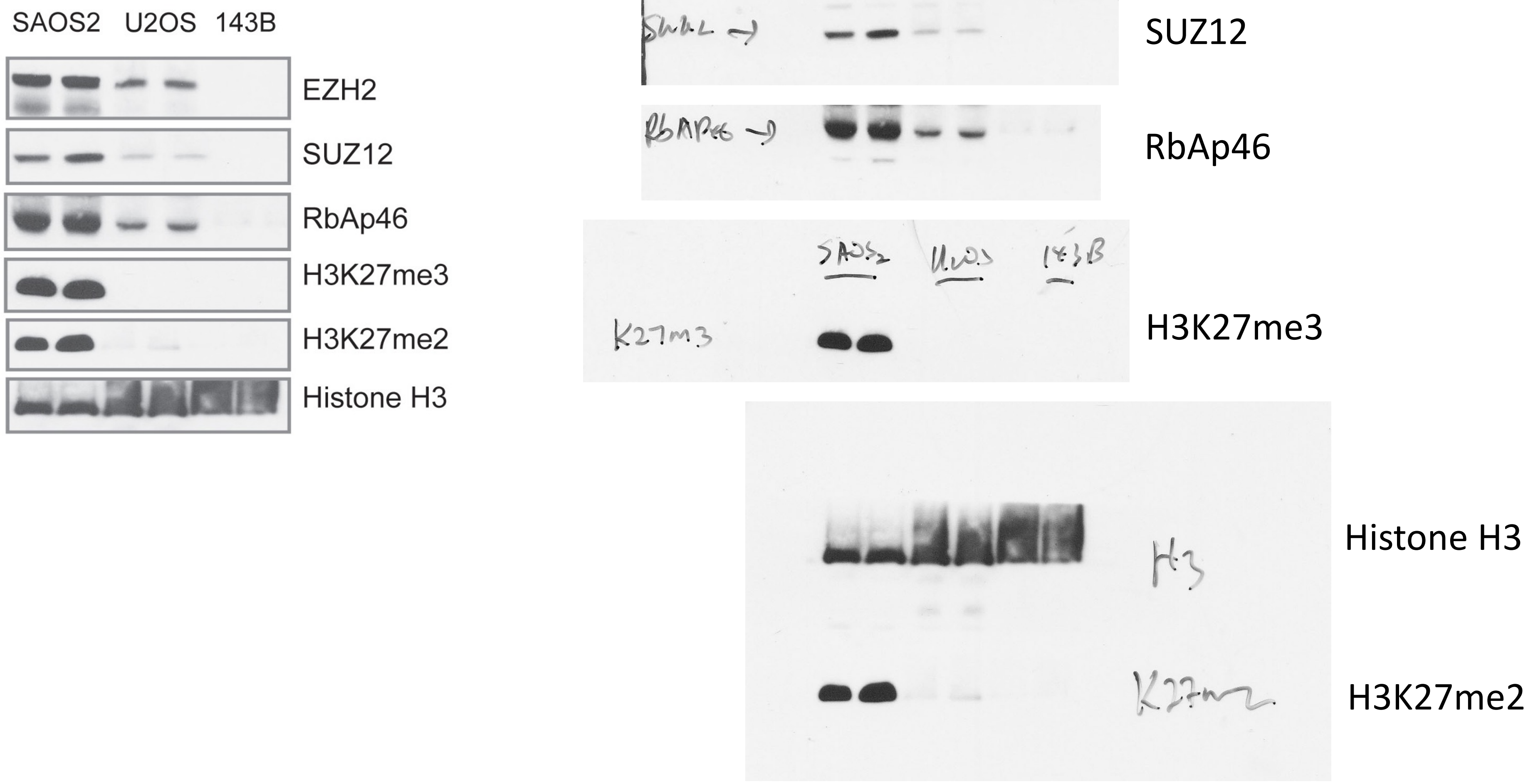
SAOS2
U2OS
143B
EZH2
SUZ12
RbAp46
H3K27me3
H3K27me2
Histone H3
EZH2
SUZ12
RbAp46
H3K27me3
Histone H3
H3K27me2
